# Automatic, machine‐agnostic, convolution‐based beam, and fluence modeling for Monte Carlo independent dose calculation

**DOI:** 10.1002/mp.17822

**Published:** 2025-04-14

**Authors:** Mingli Chen, Jingying Lin, Yang Park, Mu‐Han Lin, Arnold Pompos, Andrew Godley, Weiguo Lu

**Affiliations:** ^1^ University of Texas Southwestern Medical Center Dallas Texas USA

**Keywords:** beam modeling, monte carlo dose, independent dose

## Abstract

**Background:**

Monte Carlo (MC)‐based independent dose calculation is increasingly sought after for plan‐ and delivery‐specific quality assurance (QA) in modern radiotherapy because of its high accuracy. It is particularly valuable for online adaptive radiotherapy, where measurement‐based QA solutions are impractical. However, challenges related to beam modeling, commissioning, and plan/delivery‐specific fluence calculation have hindered its widespread clinical adoption.

**Purpose:**

We propose a generic, automated, convolution‐based beam and fluence modeling method for MC dose calculation, assuming zero or very limited knowledge of the linear accelerator (LINAC) head, with all necessary information derived from water phantom measurements. Instead of conventional particle transport through beam modulation devices (the phase space‐based approach), we developed a direct convolution‐based method to model the effects of beam modulation devices on output factors and fluence for downstream particle transport in the patient's body.

**Methods:**

The measurement data necessary for the beam model include the percent depth dose (PDD) profile of a reference field (typically 10 × 10 cm^2^), the diagonal profile of the largest field at the depth of maximum dose, and the output factors for representative field sizes formed by beam modulation devices (jaws/MLCs). The beam modeling process involves adjusting the energy spectrum to match the reference field PDD, optimizing the weighting factor for electron contamination, and encoding the output factors in a fluence convolution kernel. The fluence is calculated by convolving the intensity map defined by beam modulation devices and monitor units with the kernel, and the dose is calculated through a point source model with initial particles sampled from the fluence. This approach was demonstrated using an in‐house developed general‐purpose MC dose engine for various clinical LINACs, including those integrated with magnetic resonance imaging.

**Results:**

Compared to reference beam data, our calculations achieved average gamma passing rates of over 97% using the 2%/2 mm criteria. Compared to a sample of 20 clinical plans calculated by the treatment planning systems (TPS) across different beam modalities and treatment machines, our calculated dose achieved gamma passing rates of over 97% using the 3%/2 mm criteria with an average calculation time of less than 1 min.

**Conclusions:**

The proposed machine‐agnostic, convolution‐based beam, and fluence modeling approach enabled efficient automatic commissioning for a wide range of clinical external photon beam machines. The fluence map‐based dose calculation approached sub‐minute dose calculation efficiency for arbitrary treatment plans. The proposed method has the potential to accelerate the adoption of MC calculation‐based QA for online adaptive radiotherapy.

## 1 INTRODUCTION

Independent verification of plan and delivery dose has been an indispensable part of plan quality assurance (QA) in radiotherapy. Traditionally, it is conducted with point dose calculation and measurement‐based verification.^1^, ^2^ A thorough in silico version, independent dose calculation, has emerged as a critical component in plan QA, responding to the escalating plan complexities and a growing number of plans enabled by machine versatility and planning efficiency advancements of the treatment planning system (TPS).^3^ Traditional point dose and measurement‐based QA cannot adequately address the requirements for comprehensive, timely dose verification in the face of increasing clinical workloads, especially with online adaptive radiotherapy.^4^

Common implementations for independent dose calculations include factor‐based methods using the product of ratios^5^ and model‐based methods, such as collapsed cone convolution/superposition (CCCS) and Monte Carlo (MC) methods.^3^ The factor‐based methods have the advantage of speed but limited accuracy near field edges, in buildup regions, and for tissue heterogeneity.^3^ In contrast, model‐based dose calculations offer an evident accuracy advantage over the factor‐base calculations, especially with the precise tissue heterogeneity modeling capability of the MC method. With advancements in algorithms and GPU implementations, the speed of model‐based dose calculations has substantially improved,^6^, ^7^, ^8^, ^9^ and MC calculations, once considered impractical for clinical use, are increasingly viable for routine clinical implementations.

However, a major challenge in utilizing MC dose calculations lies in modeling the treatment beam, commissioning the model, and simulating particle transport through various beam modulation devices (jaws, multi‐leaf collimators or MLCs, blocks, wedges, etc.) across numerous segments defined by control points in a treatment plan.^10^ Here a segment refers to an intensity map shaped by the positions of the beam modulation devices and scaled by the change of monitor units (MU), with both positions and MU recorded in the control points.^10^ Note that segments can be used to depict both the static delivery as in step‐and‐shoot and dynamic MLC delivery, as the delivery information is discretized within the plan. In dynamic MLC delivery, a plan may consist of hundreds of segments, whereas in step‐and‐shoot delivery, there are typically tens of segments or less.

In the literature, treatment beam modeling is typically categorized into three types: direct simulations, phase space‐based beam modeling, and measurement‐based beam modeling.^11^ Direct simulation involves transporting particles through the linear accelerator (LINAC) head, requiring detailed material and geometric information for each component.^12^ This method is resource‐intensive and may be impeded by proprietary treatment head details, which can be difficult to obtain and subject to uncertainties in key parameters necessary for accurate simulartions.^10^, ^11^ The generated particles through the head components can be saved in phase space files for later dose calculation to reduce the repetition of simulation work. However, these phase files are large, often tens of gigabytes, requiring significant storage and lengthy loading times.^11^, ^13^ Thus, the second type of beam modeling, phase space modeling, has been employed to mitigate these issues. In this approach, beam modeling attempts to reproduce the phase space without needing to track particles through the head components or store the phase space.^14^ The third type is measurement‐based beam modeling. In this approach, the beam model is utilized to generate energy fluence, from which particles are sampled, and the attempt is to match the calculated dose with measurements while bypassing the need for extensive knowledge of LINAC head details and phase space files.^15^

Both phase space‐based and measurement‐based beam modeling utilize similar formulations based on beam characteristics derived from the phase space^14^ and are collectively referred to as virtual source modeling.^15^, ^16^ The former focuses on tuning or optimizing models to reproduce phase space data, while the latter tunes or optimizes models to match calculated dose with measurements. This measurement‐based modeling approach offers a potential advantage over other types by facilitating the application of MC dose calculations to a broader range of clinical LINACs, as measurement‐based modeling does not depend on proprietary treatment head details or cumbersome phase space files, making it ideal for independent dose verification.

For beam modeling and commissioning, the challenges stem from diverse model formulations and parameter tuning within a non‐convex setting. The challenges that plan‐specific fluence calculation poses are mainly in modeling the outputs for individual segments. In the literature, multiple sources have been employed to model primary and scatter fluence, each with a unique set of parameters and a weighting factor.^15^, ^17^ While this approach enhances model flexibility to fit the beam data, it also amplifies the complexity of model tuning and fluence calculation. Moreover, despite improvements in fitting standard field measurement beam data, achieving accurate plan‐specific fluence may remain a persistent challenge.^18^ Additionally, calculating plan‐specific fluence using direct particle transport can be very time‐consuming, particularly for dynamic deliveries involving hundreds of segments. In this process, particle interactions are simulated and tracked for each segment, with most particles being blocked, leading to reduced simulation efficiency.^19^ Therefore, various approximations have been applied to simplify the particle transport through MLC.^10^, ^19^, ^20^

**FIGURE 1:**
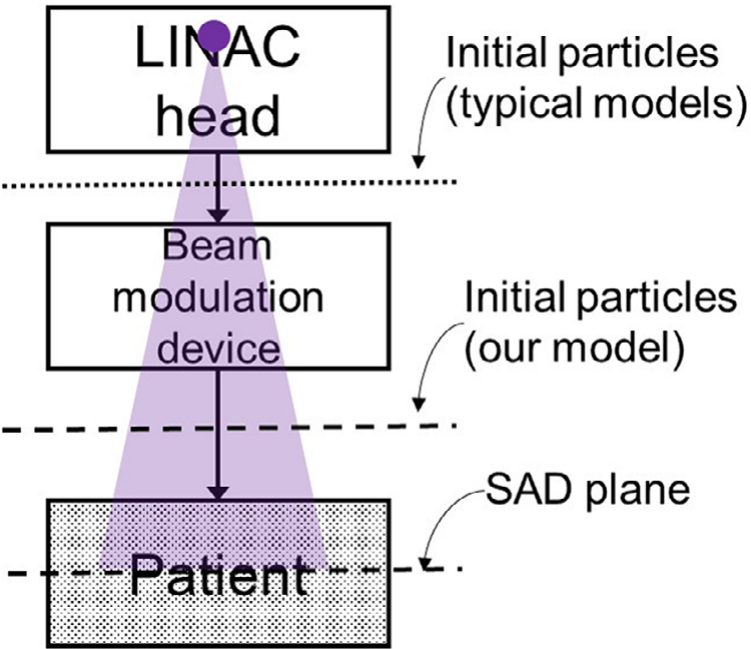
Illustration of the point source (solid circle) and radiation beam (shaded triangle) in the treatment geometry. The radiation beam is generated in the LINAC head block, goes through plan‐specific beam modulation devices, and then impinges on the patient. In our model, fluence calculation is performed on the plane at the source‐to‐axis distance (SAD), where the positions of beam modulation devices are defined. The initial particles are sampled from the fluence and back projected to a position above the patient surface and below the beam modulation devices. In contrast, typical models start the particle transport above plan‐specific beam modulation devices.

In this work, we present a single recipe of beam modeling driven by measurements for various machines, using a direct convolution‐based fluence calculation approach to address these challenges. Our model utilizes a point source with initial particles sampled from the fluence calculated on the plane at the source‐to‐axis distance (SAD) and back‐projected to a position below the plan‐specific collimators (MLC) and above the patient surface for particle transport, in contrast to typical models that initiate particle transport above the MLC (Figure 1). The fluence calculation encodes the output within a unified beam model framework, enabling streamlined, automated modeling and commissioning. We demonstrate our approach using an in‐house developed, general‐purpose MC dose engine verified previously for particle transport in patients^21^ and refer to it as the machine‐agnostic Monte Carlo (MAMC) dose calculator.

## 2 METHODS

In this section, we delineate the beam data utilized for beam modeling, provide a step‐by‐step account of constructing the beam model and fluence calculation, and describe the overall workflow for beam model commissioning. In addition, we include a subsection on magnetic field modeling for patient dose calculation, specifically for treatment machines capable of delivering beams in a magnetic field. It is important to note that fluence is impacted by various factors, and the order of beam modeling steps is crucial to untangle these influences. Throughout the beam modeling process, at each step, we tune the model parameters, calculate fluence, and sample particles from the fluence map (Figure 1) for downstream transport using our in‐house MC dose engine. The model begins with default parameters, which are then adjusted to match the calculated dose with the beam commissioning data. Readers may refer to the subsections on fluence calculation and the commissioning workflow for an overview of the entire beam modeling process while following individual steps.

### 2.1 Data for beam modeling

The following data are used for beam modeling: (1) the percent depth dose (PDD) for the 10 × 10 cm^2^ field, (2) the diagonal profile of the largest open field at the depth of maximum dose, and 3) the output factors for an array of square fields measured at a reference depth $d_{ref}$, typically including 2 × 2, 3 × 3, 5 × 5, 10 × 10, 20 × 20 cm^2^, etc. The reference depth is the depth at which machine outputs are defined and measured during commissioning, and it varies between machines. These data are typically part of the commissioning beam measurements for treatment machines. Below, we refer to the commissioning beam data measurements as the reference beam data.

### 2.2 Beam modeling

The beam model is constructed by explicitly modeling the following components: (1) X‐Ray Spectrum, (2) electron contamination, (3) open field intensity map, and (4) head scatter factor (Sc), in this specific order.

#### 2.2.1 Beam modeling MC simulation setup

For beam modeling, we use MC calculation on a water phantom with the same setup conditions (SAD, SSD, field sizes) as the corresponding measurements. For field sizes ≥ 5 × 5 cm^2^, a phantom size of 50 × 50 × 50 cm^3^ with a voxel size of 0.2 × 0.2 × 0.2 cm^3^ is used. For field sizes less than 5 × 5 cm^2^, a phantom size of 10 × 50 × 10 cm^3^ with a voxel size of 0.1 × 0.1 × 0.1 cm^3^ is used. The number of particles used for each calculation is 10^10^ for field sizes ≥ 10 × 10 cm^2^, and the number is 10^9^ for smaller field sizes.

#### 2.2.2 Tuning the spectrum

**FIGURE 2:**
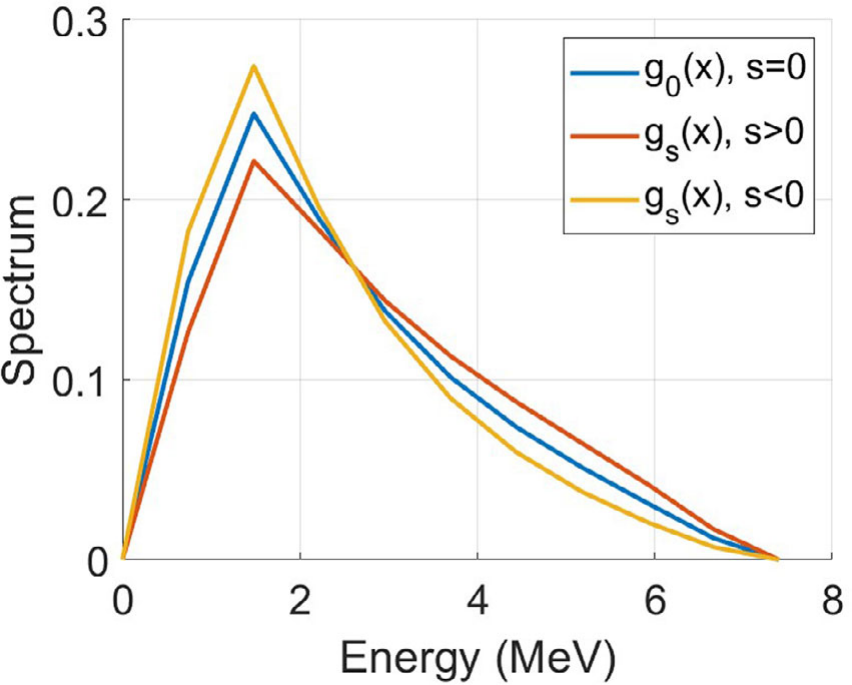
Illustration of the effect of the tuning parameter s defined in Equation (1) on the beam energy spectrum.

The first step in beam modeling is to tune the spectrum. From an initial spectrum $g_{0}\left(E\right)$, which can be a spectrum for any common LINAC of a similar nominal beam energy,^22^ a single scale factor of slope s is applied to make the adjusted spectrum $g_{s}\left(E\right)$ harder or softer (Equation 1). Here, E denotes the energy, and $\bar{E}={\left\langle E\right\rangle }_{g_{0}}$ denotes the mean energy. By increasing the slope s, it increases the distribution for $E>\bar{E}$ and decreases the distribution for $E<\bar{E}$, that is, making the beam harder. Similarly, decreasing the slope s makes the beam softer. It is straightforward to verify that the integral of the adjusted spectrum remains equal to one $\int g_{s}\left(E\right)dE=1$ if the integral of the initial spectrum is one $\int g_{0}\left(E\right)dE=1$. Since the changes of slope are small, the multiplicative factor applied to $g_{0}\left(E\right)$ in Equation (1) remains positive. The behavior of the tuning parameter and the adjusted spectrum is illustrated in Figure 2.

(1) $$g_{s}\;\left(E\right)=g_{0}\;\left(E\right)\cdot \left(1+s\left(E-\bar{E}\right)\right)$$

Note that after tuning, the mean energy is ${\left\langle E\right\rangle }_{g_{s}}$.

$$\begin{array}{ccc} & & {\langle E\rangle }_{g_{s}}=\int Eg_{s}\left(E\right)dE=\int E\left(g_{0}+sEg_{0}-s\bar{E}g_{0}\right)dE\\ & & ={\langle E\rangle }_{g_{0}}+s{\langle {\left(E-\bar{E}\right)}^{2}\rangle }_{g_{0}} \end{array}$$

The adjusted spectrum that yields the closest match between the PDD extracted from the MC dose $D(g_{s},z)$ and the measured PDD, based on the mean square deviation for the reference field (typically 10 × 10 cm^2^), is selected for the beam model (Equation 2). Here z denotes the depth variable.

(2) $$\hat{s}=\arg\min_{s}\int {\left(D\left(g_{s},z\right)-PDD_{meas}\left(z\right)\right)}^{2}dz\;$$

#### 2.2.3 Modeling electron contamination

Electron contamination primarily occurs with the high energy beams and mainly affects the dose distribution at shallow depths, $d\leq d_{max}$.^23^, ^24^ To account for this, we use the portion of the PDD in the buildup region to adjust the weighting factor for electron contamination. Specifically, we calculate the photon component $D_{x}$ for the 10 × 10 cm^2^ field using the energy spectrum obtained in the first step and compare it to the measured PDD. The discrepancy between $D_{x}$ and PDD in the buildup region is attributed to the electron contamination, $w_{e}D_{e}$, as expressed in Equation (3). Here, $D_{e}$ is the dose calculated by sampling the electron fluence (assuming the same energy fluence as photons) and normalized to its maximum, and $w_{e}$ is the weighting factor to be tuned.

$$D\;\left(z\right)=D_{x}\;\left(z\right)+w_{e}\cdot D_{e}\left(z\right)$$

(3) $${\hat{w}}_{e}=\arg\min_{w_{e}}{∥D\left(z\right)-PDD_{meas}\left(z\right)∥}_{z\leq d_{max}}^{2}\;$$

#### 2.2.4 Open field intensity map

The open field intensity map, also called the off‐axis scale (OAS) map, is the fluence of the largest field normalized to the center. We use the diagonal profile h of the largest field at $d_{max}$ to model the initial OAS $C_{0}$.

(4) $$C_{0}\;\left(x,y\right)=h\left(r\right)\text{, where}\;r=\sqrt{x^{2}+y^{2}}\;$$

The calculated dose profile $h_{0}$ based on the initial map $C_{0}$ is incorporated in the OAS update: $C_{1}=C_{0}\;\cdot \left(h/h_{0}\right).$ Both the OAS and the profile h are unitless, as they are normalized to the center (origin).

#### 2.2.5 Modeling in‐air output factors $S_{c}$

The in‐air output factor (or collimator scatter) $S_{c}$ is typically measured experimentally using a miniphantom.^23^, ^25^ In our approach, we model the effect of collimator scatter using a fluence convolution kernel, equivalent to representing the collimator scatter as a point spread function. We derive the collimator scatter in a self‐consistent manner using output factors measured in water at a reference depth $d_{ref}$. These output factors are the total scatter factor $S_{cp}$, which represents the composite effect of both collimator scatter ($S_{c}$) and phantom scatter ($S_{p}$). To extract $S_{c}$, we first calculate the dose for various field sizes at $d_{ref}$ without including collimator scatter by omitting the application of fluence convolution. The differences in dose values are attributed to the phantom scatter $S_{p}$, as it is modeled in the MC dose calculation. Then, we divide $S_{cp}$ by $S_{p}$ to determine the collimator scatter, that is, $S_{c}=S_{cp}/S_{p}$. Next, to derive the convolution kernel K for collimator scatter, we apply our model that the convolved fluence at the center equals to $S_{c}$ of corresponding collimated fields, yielding an integral equation as expressed in Equation (5). In this equation, K denotes the kernel, C denotes the OAS map, and $\left(f_{x},f_{y}\right)$ denotes the field size.

(5) $$S_{c}\;\left(f_{x},f_{y}\right)=\int_{-f_{y}/2}^{f_{y}/2}\int_{-f_{x}/2}^{f_{x}/2}K\left(x,y\right)C\left(x,y\right)dxdy\;$$

That is, the convolution kernel K is the derivative of $S_{c}$ divided by the OAS C, as in Equation (6). In computation, we employ scatter data interpolation to obtain a 2D map of $S_{c}$ and apply the difference function instead of differentiation to derive K.

(6) $$K\;\left(x,y\right)=\frac{d^{2}S_{c}\left(x,y\right)}{dxdy}/C\left(x,y\right)\;$$

### 2.3 Dose calculation

The dose calculation steps involved (1) calculating beam/segment intensity, (2) applying open field intensity, (3) convolution with the scatter kernel, (4) weighting by energy spectrum, and 5) particle sampling and transport in the patient by the MC dose engine, where steps (1)–(3) are fluence calculation as given in Table 1. The resolution of the fluence map was 1 × 1 mm^2^, and the dose calculation grid resolution was 2 × 2 × 2 mm^3^. Photons and electrons were sampled from the calculated energy fluence distribution using the Metropolis‐Hastings sampling algorithm for efficiency^8^, ^26^ and then transported by the MC dose engine with a total of 10^9^ initial photons and 10^7^ initial electrons. The final dose was their weighted sum using the weight in Equation (3). The energy transport included magnetic field modeling to account for treatment machines equipped with magnetic resonance imaging.

**TABLE 1:** Summary of fluence calculation steps.

1. Calculate segment intensity. $I\;\left(x,y\right)=\sum_{i}M_{i}T_{i}\left(x,y\right)$ i, the segment index, $M_{i}$, the monitor units for the ith segment, $S_{i}$, the aperture shape for the ith segment, i.e., $S_{i}\;\left(x,y\right)=\left\{\begin{array}{c} 1,\;(x,y)\;is\;not\;blocked\;by\;the\;beam\;modulation\;device\\ 0,\;otherwise\; \end{array}\right.$ $\;T_{i}=\left(S_{i}\setminus \partial_{y}S_{i}\right)+\alpha \left(\partial_{y}S_{i}\right)+\beta \left(1-S_{i}\right)$, intensity correction with default values α=0.75 and β=0. 2. Scale by the OAS map C in 2.2.4. I(x,y)←I(x,y)·C(x,y) 3. Calculate the fluence to account for collimator scatter. f(x,y)=(I⊗K)(x,y), where K is the collimator scatter kernel in Equation (6).

#### 2.3.1 Fluence calculation

For fluence calculation, we used the segment information from the TPS, including the MUs, MLC leaf positions, and beam angles. Let us assume that there is only one beam angle since fluence is calculated separately for each beam angle in the beam eye's view (BEV) coordinates.^9^ Let $M_{i}$ denote the MU for the ith segment, $S_{i}\left(x,y\right)$ denote the aperture shape defined by leaf positions, and $T_{i}\left(x,y\right)$ denote intensity correction for the tongue and groove effect and leakage.^27^, ^28^, ^29^ The tongue and groove effect is modeled as reduced intensity at the segment boundary along the y‐direction,^30^ $\partial_{y}S_{i}$, assuming leaf motion in the x direction, with the reduced factor α having a default value of 0.75. Leakage, including transmission and leaf tip effects, is modeled outside the field, indicated by $1-S_{i},$ with the leakage factor β set to a default value of 0. Thus, $T_{i}$ is one everywhere within $S_{i}$, except at the field boundary $\partial_{y}S_{i}$, where it takes the value α, and outside the field $1-S_{i}$, where it takes the value β (Table 1).

The intensity of all segments can be expressed as $I\;\left(x,y\right)=\sum_{i}M_{i}T_{i}\left(x,y\right)$, where x and y are the coordinates on the SAD plane (Figure 1). Next, we applied the OAS map C described in 2.2.4 to the segment intensity I: I(x,y)←I(x,y)·C(x,y). Then, we convolve the scaled segment intensity by the scatter kernel in Equation (6) to obtain fluence f=I⊗K. The fluence calculation steps are summarized in Table 1. After that, we weight the fluence f by the energy spectrum g(E) to obtain the energy fluence $f_{g}$. Now we can employ the MC dose engine to calculate patient dose $D=MC(\rho \left(x,y,z\right),f_{g}\left(x,y\right))$. Here ρ denotes the patient density, g denotes the energy spectrum, and x, y, and z denote the spatial coordinates. The particles for patient transport are sampled from $f_{g}\left(x,y\right)$. The calculation steps apply to both dose $D_{x}$ of photon fluence and dose $D_{e}$ of electron fluence. The final dose is the weighted sum of the two components, $D=D_{x}+w_{e}\cdot D_{e}$.

#### 2.3.2 Modeling the magnetic field

We extended our in‐house GPU‐based Monte‐Carlo simulation package to include charged particle transport in external static magnetic fields. An arbitrary 3D magnetic field was modeled with three volumes, $B_{x}$, $B_{y}$, and $B_{z}$, of the same dimensions as the CT volume, the trajectory deflection was calculated with relativistic Lorentz force at every simulation step, and the magnetic field was tri‐linearly interpolated. Specifically, let v denote the electron motion direction without the magnetic field. Let ${\vec{v}}^{\prime }$ denote the deflected motion direction under the influence of the magnetic field $\vec{B}$. The deflected electron's motion is $s{\vec{v}}^{\prime }$, where s is the step size sampled in condensed history. The amount of deflection $s\Delta v=s{\vec{v}}^{\prime }-s\vec{v}$ can be calculated using similar triangles in the first order approximation $\Delta v=\left(s/R\right)\;\left(\vec{v}\times \vec{B}/∥\vec{v}\times \vec{B}∥\right)$ and R is the circle of deflection due to the relativistic Lorentz force.

### 2.4 Commissioning and validations

**FIGURE 3:**
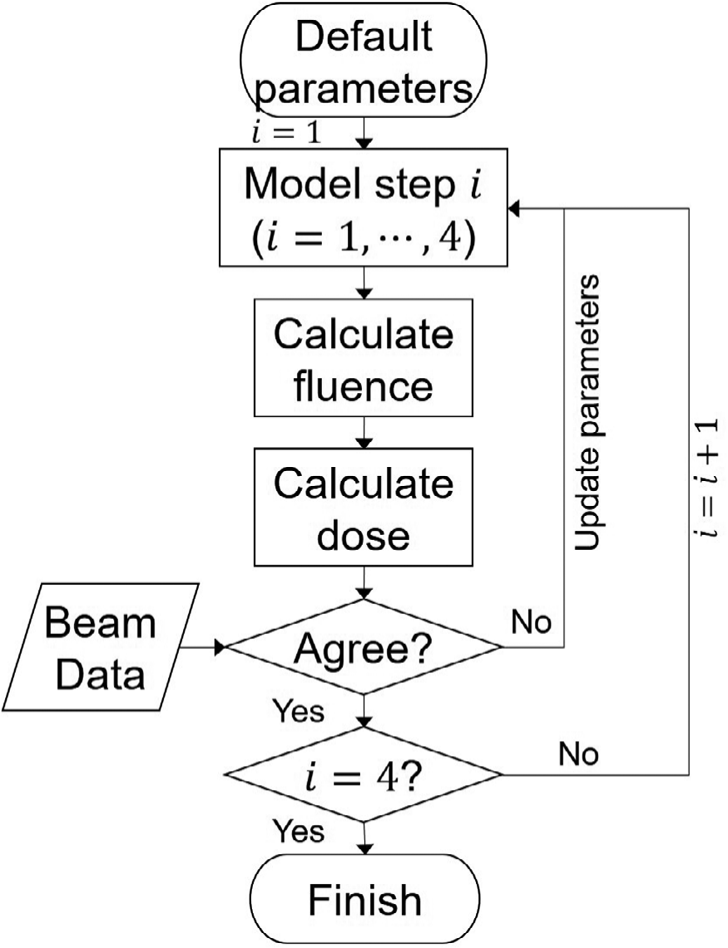
Beam model commissioning flowchart. There are four modeling steps: (1) tuning the spectrum, (2) tuning the weighting factor for electron contamination, (3) tuning the OAS, and (4) constructing the fluence convolution kernel for collimator scatter. Model parameters are adjusted to achieve the closest match between calculated and measured beam data.

Commissioning essentially involves revisiting the steps in beam modeling and dose calculation, with a primary emphasis on parameter tuning (Figure 3). This process entails adjusting the parameters to achieve the closest match between the calculated dose and measured data. The parameter tuning adheres to the order described in beam modeling: (1) tuning the spectrum; (2) tuning the weighting factor for electron contamination; (3) tuning the OAS; (4) constructing the fluence convolution kernel for collimator scatter. This processing sequence is designed in this order because factors in the earlier steps are less dependent on those in the later steps. Spectrum tuning is designated as the first step because it is the least dependent on other factors.

The calculations were compared to the measurement beam data and clinical plans generated by the commercial TPS across different beam modalities, including 6X, 10X, 15X, 6XFFF, 10XFFF; machines, including Varian's True‐Beam (TB), Vital‐Beam (VB), Halcyon, and Ethos, and Elekta's Versa and Unity‐7X; and delivery techniques, including 3D (conformal), IMRT (static beam and dynamic MLC), VMAT, and SS (step and shoot). The TPS used was either Eclipse, which utilizes the AAA or Acuros XB dose calculation algorithms,^30^ or Monaco, which employs MC dose calculation.^31^ Our calculations were conducted on an Intel Xeon Gold 5215 CPU @ 2.50 GHz and an NVIDIA Tesla V100‐PCIE‐32GB GPU.

## 3 RESULTS

### 3.1 Energy spectra, fluence convolution kernels

**FIGURE 4:**
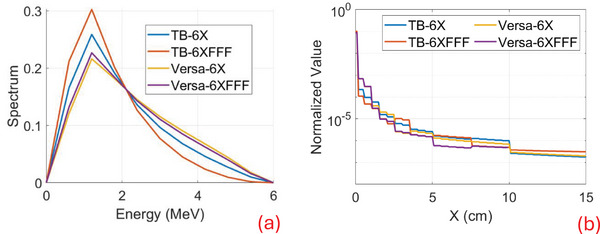
Beam model parameters for TB‐6X, TB‐6XFFF, Versa‐6X, and Versa‐6XFFF. (a) Energy spectrum. (b) Fluence convolution kernel in log scale plot.

Following the steps and workflow described in Section 2, we automated the beam modeling process. Below, we present the energy spectra and fluence convolution kernels for the beam model parameters of TB‐6X, TB‐6XFFF, Versa‐6X, and Versa‐6XFFF (Figure 4). The flattened beams (TB‐6X and Versa‐6X) are slightly harder than their flattening‐filter free counterparts (TB‐6XFFF and Versa‐6XFFF), and Versa beams are harder than those of TB, as shown in Figure 4a. The fluence convolution kernels are displayed in Figure 4b on a logarithmic scale, as they drop sharply from the center, and are shown only on the positive x‐axis, as they are circularly symmetric.

### 3.2 Beam data

**FIGURE 5:**
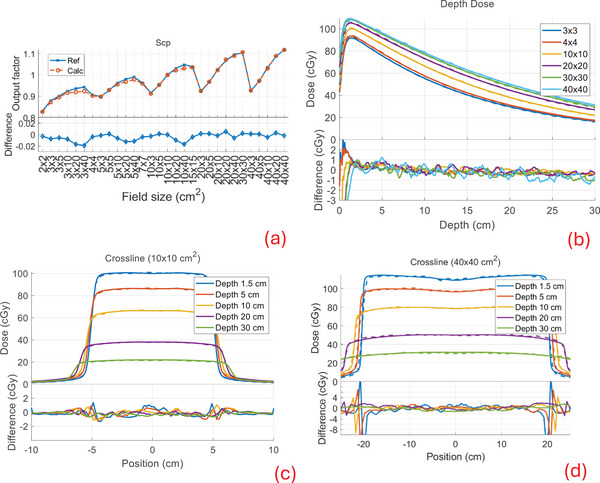
Beam data comparison between calculation (dashed) and the reference (solid) for TB‐6X. (a) Output factors (Scp) for various fields. (b) Depth dose profiles for various square fields. (c) Cross‐profiles for the 10 × 10 cm^2^ field at various depths. (d) Cross‐profiles for the 40 × 40 cm^2^ field at various depths. The curves at the bottom in each subfigure (a)–(d) are the difference between the calculation and the reference (calc‐ref).

**FIGURE 6:**
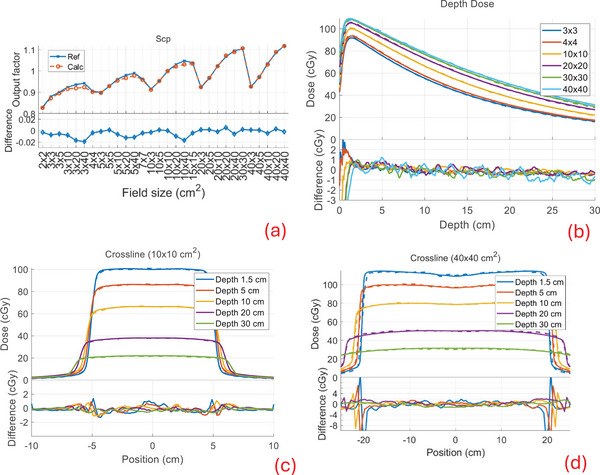
Beam data comparison for TB‐6XFFF. See Figure 5 for captions (a)–(d).

**FIGURE 7:**
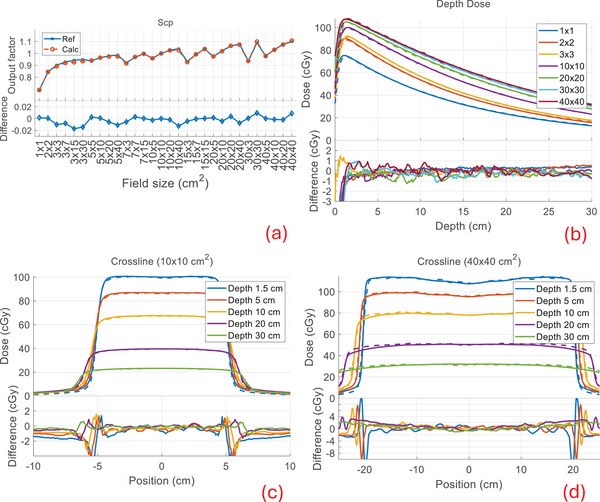
Beam data comparison between calculation (dashed) and the reference (solid) for Versa‐6X. See Figure 5 for captions (a)–(d).

**FIGURE 8:**
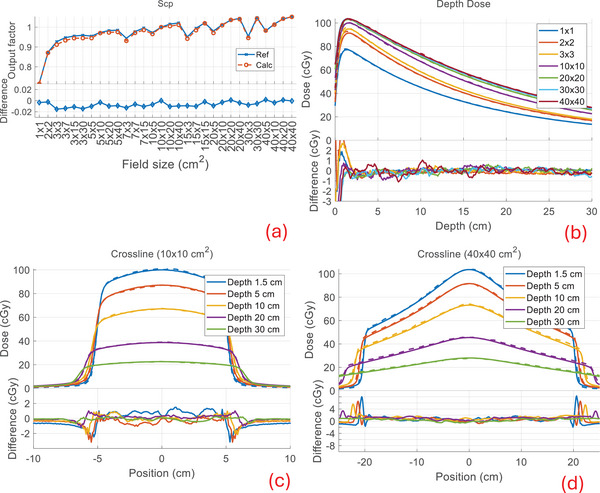
Beam data comparison for Versa‐6XFFF. See Figure 5 for captions (a)–(d).

Our calculation compared well to the measured beam data with similar performance for all combinations of modalities and machines available in our clinic. Below, we show the comparison of the output factors ($S_{cp}$), depth dose profiles, and cross profiles between our calculation and measurements (denoted as reference) for TB‐6X, TB‐6XFFF, Versa‐6X, and Versa‐6XFFF in Figures 5, 6, 7, 8. The output factors were calculated/measured at the reference depth and normalized to the 10 × 10 cm^2^ field. The dose profiles were shown in percentage dose values relative to the central value of the 10 × 10 cm^2^ field at the depth of maximum dose (1.5 cm). For the output factors, the difference between calculations and the reference were within 2% across all field sizes and modalities, as shown in subfigures (a), with an average difference of −0.1 ± 0.6% and a maximum difference of 1.7%. The depth dose deviations were within 2% for all field sizes and modalities, except in the buildup regions (≤1.5 cm), as shown in subfigures (b). The average difference beyond the buildup regions was −0.1 ± 0.4%, with a maximum deviation of 1.8%. The cross‐profiles of the 10 × 10 cm^2^ field at various depths are shown in subfigures (c) and those of the 40 × 40 cm^2^ field in subfigures (d). For the 10 × 10 cm^2^ field, the overall maximum difference was 1.7% in both the high‐ and low‐dose regions, with a maximum distance to agreement of 1 mm in the penumbra region (20%–80% of the profile center). For the 40 × 40 cm^2^ field, the overall maximum difference was 2.8% in both the high‐ and low‐dose regions, with a maximum distance to agreement of 3 mm in the penumbra region. The beam data calculations for Ethos and Unity‐7X exhibited similar performance to other FFF beams, as shown in a previous publication.^4^ The beam data for Ethos and Unity‐7X, as well as additional profiles (inline/crossline) and statistical summary, are included in Supporting Information. The calculation for all cases had an average voxel uncertainty of less than 1%.

### 3.3 Clinical plans

We present a comparison of our calculated dose $d_{MC}$ to the TPS dose $d_{TPS}$ using point dose difference and the gamma passing rate for a sample of 20 clinical plans encompassing various treatment sites and modalities (Table 2). The point dose difference was calculated as the ratio of the dose difference to the TPS dose, $\left(d_{MC}-d_{TPS}\right)/d_{TPS}$, at a reference point. This reference point (${\vec{x}}_{ref}$) was defined as the location of the maximum distance from within the 90% isodose volume $V_{90}$ to its surface $\partial V_{90}$: ${\vec{x}}_{ref}=\arg\max_{\vec{x}\in V_{90}}∥\vec{x}-\partial V_{90}∥$. The gamma criteria were 3%/2 mm, and the gamma index was evaluated for dose of 10% maximum dose or greater. The point dose difference was less than 3%, and the gamma passing rate was greater than 97% in these cases. Additionally, all calculations were completed in ∼1 min or less with average voxel uncertainty of less than 1%.

**TABLE 2:** Comparison between our dose calculation and the TPS in terms of point dose difference and the gamma passing rate. The last column shows our dose calculation time in the unit of second for each case.

Case	Site	Modality	Technique	Point dose (%)	γ pass (%)	Calc time (s)
1	Brain	Versa 6X	3D	−0.95	97.55	21
2	Pelvis	Versa 10X	VMAT	1.21	99.70	52
3	Brain	Versa 15X	IMRT	1.76	99.92	32
4	Lung	Versa 6XFFF	VMAT	−1.25	99.88	31
5	HN	TB6X	VMAT	0.54	99.62	34
6	Pelvis	TB10X	VMAT	0.63	100.00	49
7	Pelvis	TB15X	IMRT	−1.01	98.51	29
8	Thorax	TB15X|6X	VMAT|IMRT	−0.55	99.65	31
9	Spine	TB10XFFF	VMAT	0.66	100.00	45
10	Brain	VB15X	VMAT	−2.30	99.40	54
11	Breast	VB15X|6X	IMRT|3D	−0.61	98.59	24
12	Breast	VB10X|6X|15X	IMRT|3D|IMRT	−0.44	99.98	27
13	HN	Unity	SS	0.91	98.85	59
14	Pelvis	Unity	SS	−2.29	99.72	65
15	Brain	Unity	SS	0.72	99.49	42
16	Abdomen	Unity	SS	−0.38	98.56	65
17	Lung	Ethos	IMRT	0.19	99.96	29
18	Breast	Ethos	IMRT	2.32	99.95	20
19	HN	Halcyon	IMRT	0.12	99.80	21
20	Pelvis	Halcyon	VMAT	−2.66	99.71	26

**FIGURE 9:**
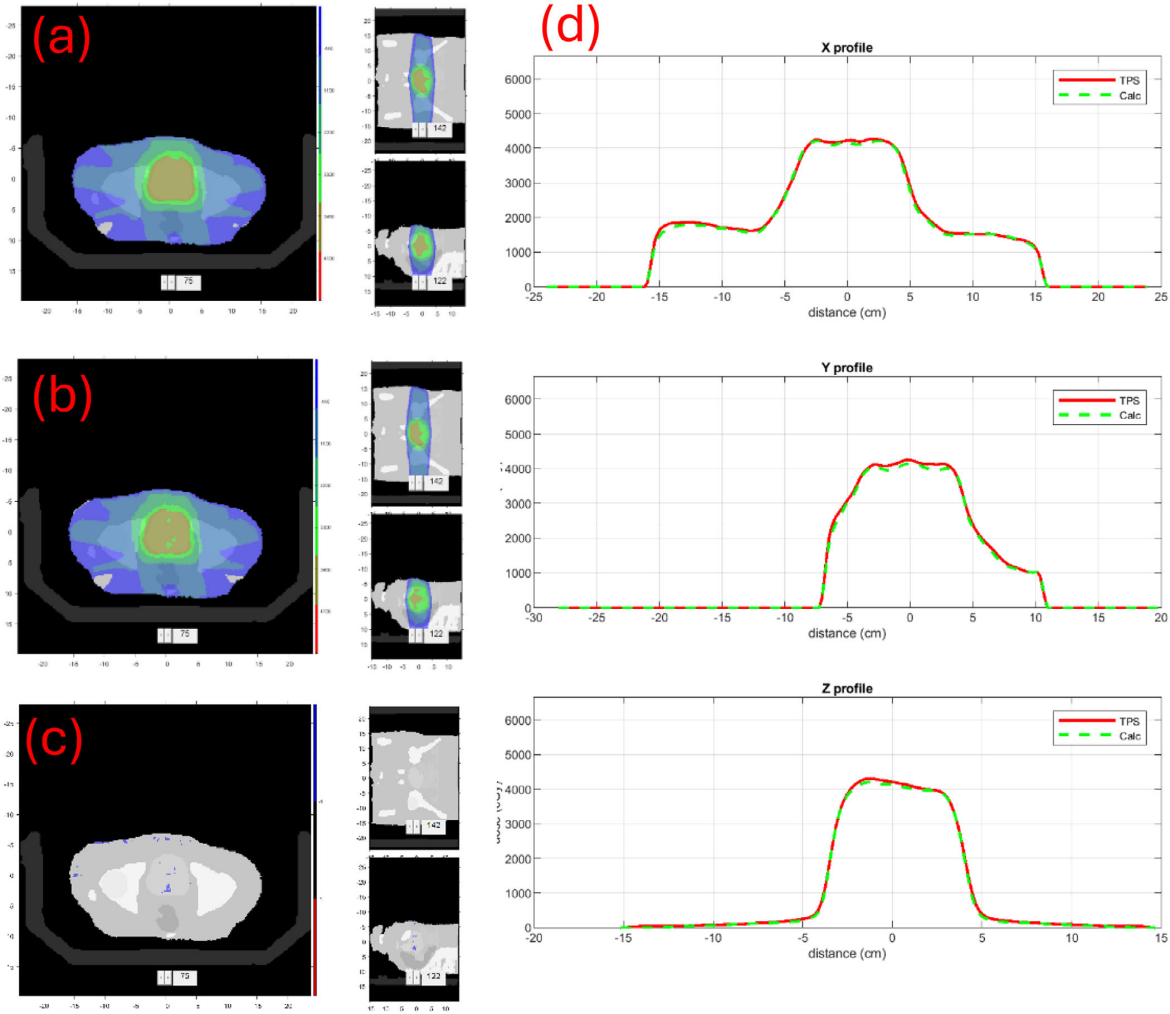
Case #14 in Table 2, a pelvis treatment site with Unity. (a) TPS dose. (b) Our MC dose. (c) Signed Gamma Index map. (d) Comparison of three orthogonal profiles (x, y, and z) between TPS (solid) and our calculation (dashed).

**FIGURE 10:**
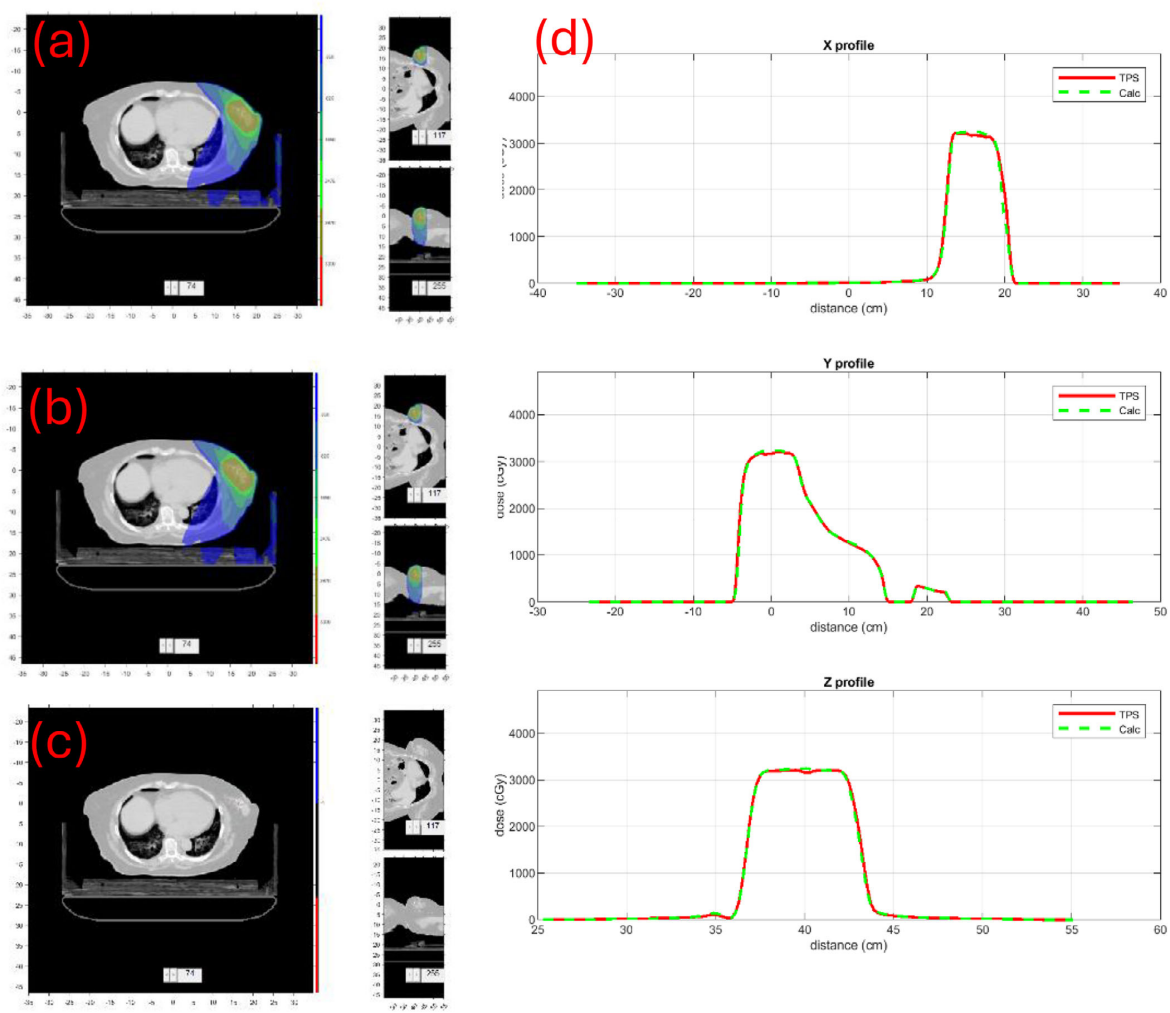
Case #18 in Table 2, a breast treatment site with Ethos. (a) TPS dose. (b) Our MC dose. (c) Signed Gamma Index map. (d) Comparison of three orthogonal profiles (x, y, and z) between TPS (solid) and our calculation (dashed).

The dose distributions for a couple of cases are illustrated. Figure 9 compares the dose distributions and three orthogonal profiles of case #14, a pelvic case treated with Unity. Figure 10 compares doses of case #18, a breast case treated with Ethos. These results indicate good agreement between our calculations and those of TPS both quantitatively and qualitatively.

## 4 DISCUSSION

Independent dose calculation requires accurate beam modeling and efficient implementation to perform plan dose verification and meet the time demand of a busy clinical workload. However, obtaining an accurate beam model is challenging and time‐consuming, especially with MC methods that involve simulating a large number of particles in an iterative process between model tuning and downstream dose calculation. Beam modeling for different treatment machines may require different model formulation with different complexity, while machine‐specific information may be unavailable to the community. Additionally, plan‐specific fluence calculation can also pose challenges as it can be time‐consuming to simulate particles for each segment and in terms of accurately producing the output for various segments. Our proposed approach addresses these challenges with the following features: (1) a single recipe for various machines and modalities, (2) a streamlined, unified modeling framework, (3) a convolution kernel to encode output factors for fluence calculation, and (4) particle transport starts below plan‐specific collimators.

It is worth noting several distinct processes of the proposed beam modeling, including modeling the electron contamination, output factors, magnetic field, and plan‐specific fluence. The electron contamination increases with increasing field size and beam energy but mainly affects the dose in shallow depth.^32^ Therefore, we used the PDD in the buildup region to optimize the weight of electron contamination. The electron transport in the MC engine used the continuous slowing down approximation^33^ with a fixed voxel resolution of 2 × 2 × 2 mm^3^. Within each voxel, the magnetic field from the MR‐LINAC can be modeled that the electron would experience the Lorentz force and result in an altered momentum. Fluence calculation utilized a scatter kernel to model the outputs and convolved it with the segment intensity that accounted for the tongue and groove effect and leakage^29^ to obtain plan‐specific fluence. The outputs that were used to derive the scatter kernel were based on the square fields of the commissioning measurement data. The fluence calculation does not involve the use of the phase space. The particles for patient transport are sampled from the fluence maps. This beam modeling approach can calculate plan‐specific fluence very efficiently with convolution and facilitate real‐time dose calculation.

This fluence calculation approach was part of the steps in beam modeling and enabled a simple recipe to untangle the mixed effects of beam properties. The recipe progressively added the more dependent factors and encoded the outputs in a distribution without needing a multiple source model. Separating and reducing the factors make model fitting straightforward and facilitate automation. Automatic commissioning is desirable as it can streamline the process and improve efficiency, while the ease and level of automation depend on the complexity of the beam model.

The proposed beam modeling approach exhibits some universal features. While it was demonstrated using an in‐house developed general‐purpose MC dose engine, it can also be adapted to other dose engines, although the beam parameters may require re‐tuning. Our testing of this beam modeling and fluence calculation approach on various treatment machines has demonstrated excellent dosimetric agreement, suggesting a form of universal beam modeling. The output‐encoded fluence convolution kernel offers a universal method for efficient plan‐specific fluence calculation. Furthermore, since the commissioning is based on measured beam data without assumptions about LINAC head configurations, the model is LINAC head agnostic. Ultimately, by not relying on the beam model of the TPS and using a different dose calculator than the TPS, this approach establishes a genuinely independent dose calculator.

## 5 CONCLUSION

We introduced a machine‐agnostic, convolution‐based approach of beam and fluence modeling for model‐based independent dose calculation. We demonstrated the approach using an in‐house developed general‐purpose MC dose calculation across diverse clinical treatment machines. This advancement facilitates efficient automatic commissioning and rapid fluence generation, potentially accelerating the adoption of MC dose calculation in clinical practice.

## CONFLICT OF INTEREST STATEMENT

The authors declare no conflicts of interest.

## Supporting information



Supporting information

Supporting information

Supporting information

Supporting information
