# Supplementary material for: Automatic, machine‐agnostic, convolution‐based beam, and fluence modeling for Monte Carlo independent dose calculation
Source: Med Phys. 2025 Apr 14;52(7):e17822. doi: 10.1002/mp.17822 (PMC12257457; doi:10.1002/mp.17822)
Supplement: Supplementary file 1 — Supporting information [file MP-52-0-s004.pdf]

Supplementary Document

This document is a supplement to the paper "Automatic, Machine-Agnostic, Convolution-Based Beam and Fluence Modeling for Monte Carlo Independent Dose Calculation." It provides additional beam data comparisons between calculations and reference, including beam data for Ethos (Figure S1) and Unity (Figure S2), diagonal profiles for TB-6X, TB-6XFFF, and Ethos (Figure S3a, b, e), inline profiles for Versa-6X, Versa-6XFFF, and Unity (Figure S3c, d, f), and a statistical summary for output factors, percent depth dose, and profile data (Tables S1–S5).

Ethos

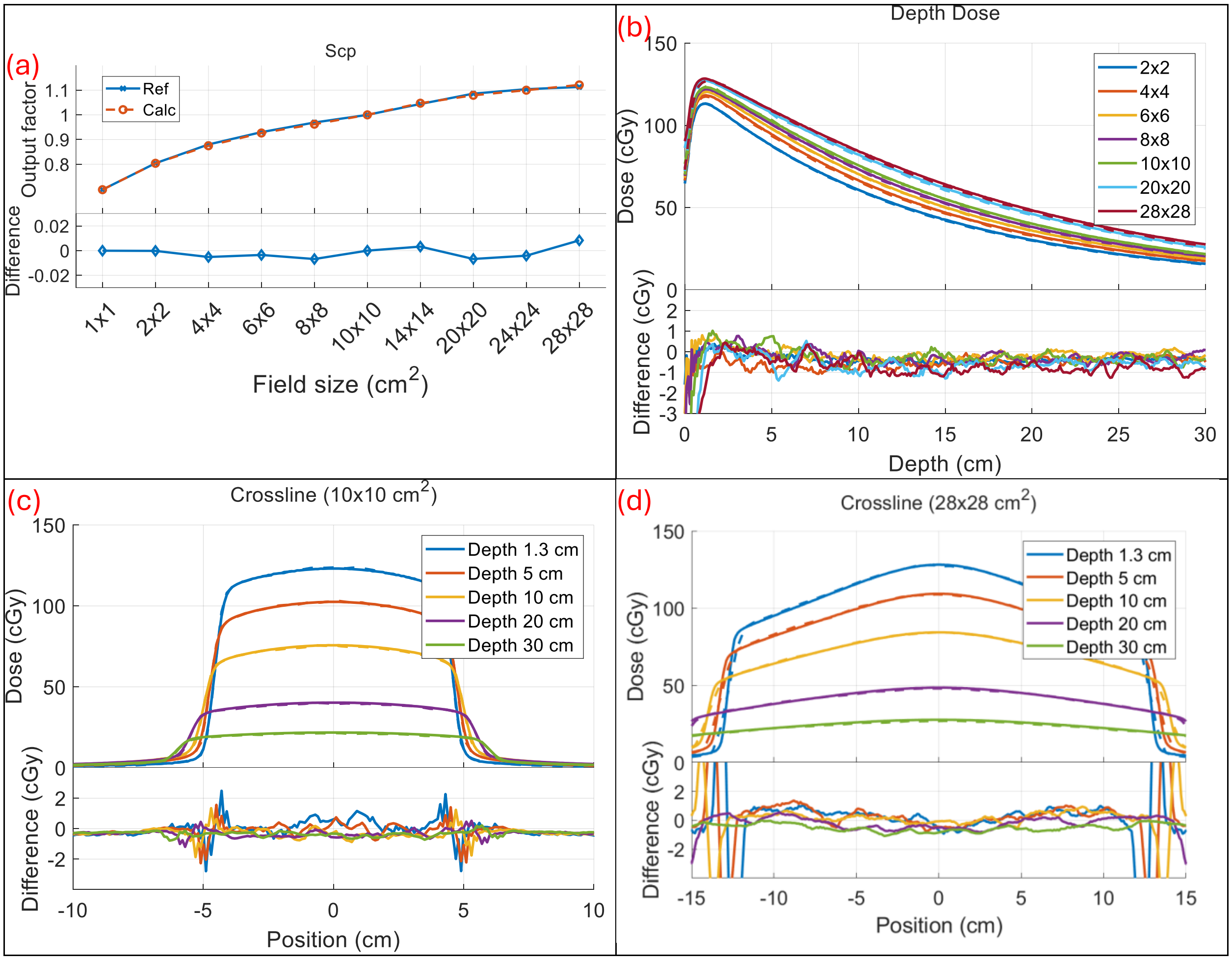

Figure S1. Comparisons of calculation (dashed) and reference beams (solid) for Ethos. (a) Scp of various field sizes. (b) Depth dose curves of various field sizes. (c) Cross profiles of the 10x10 cm^2 field at various depths. (d) Cross profiles of the 28x28 cm^2 field at various depths.

Unity

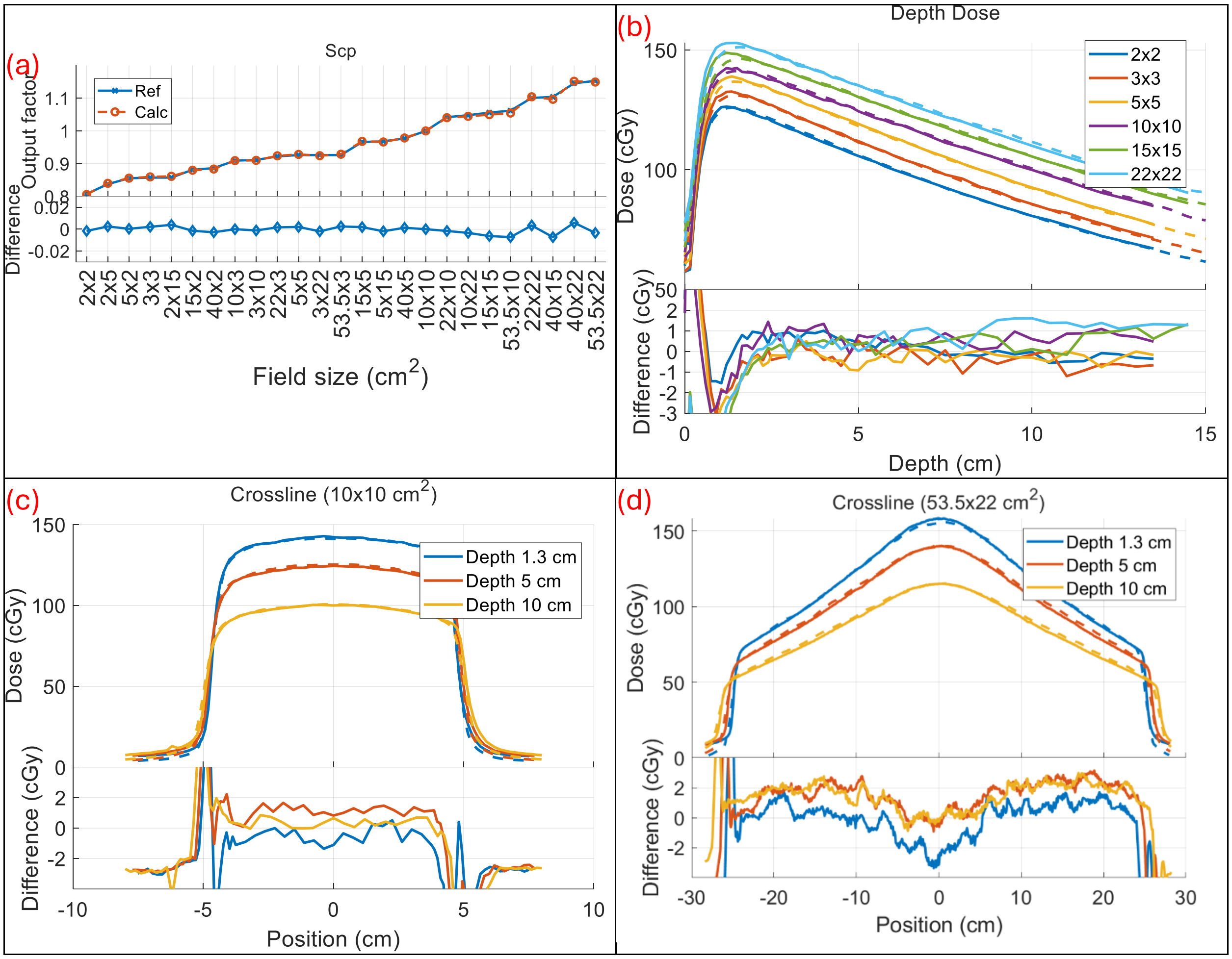

Figure S2. Comparisons of calculation (dashed) and reference beams (solid) for Unity. (a) Scp of various field sizes. (b) Depth dose curves of various field sizes. (c) Cross profiles of the 10x10 cm^2 field at various depths. (d) Cross profiles of the 53.5x22 cm^2 field at various depths.

Inline & Diagonal profiles

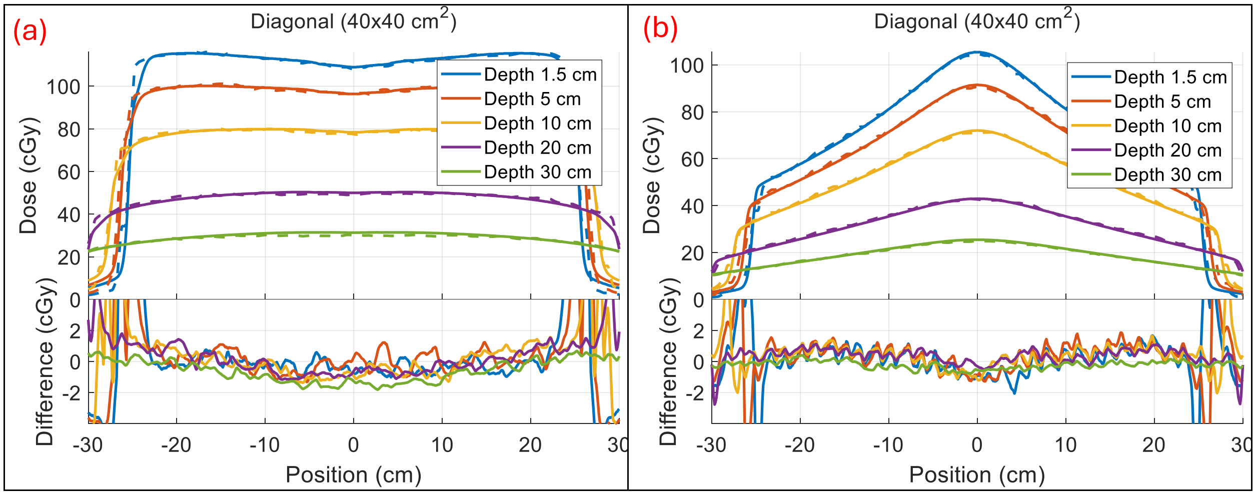

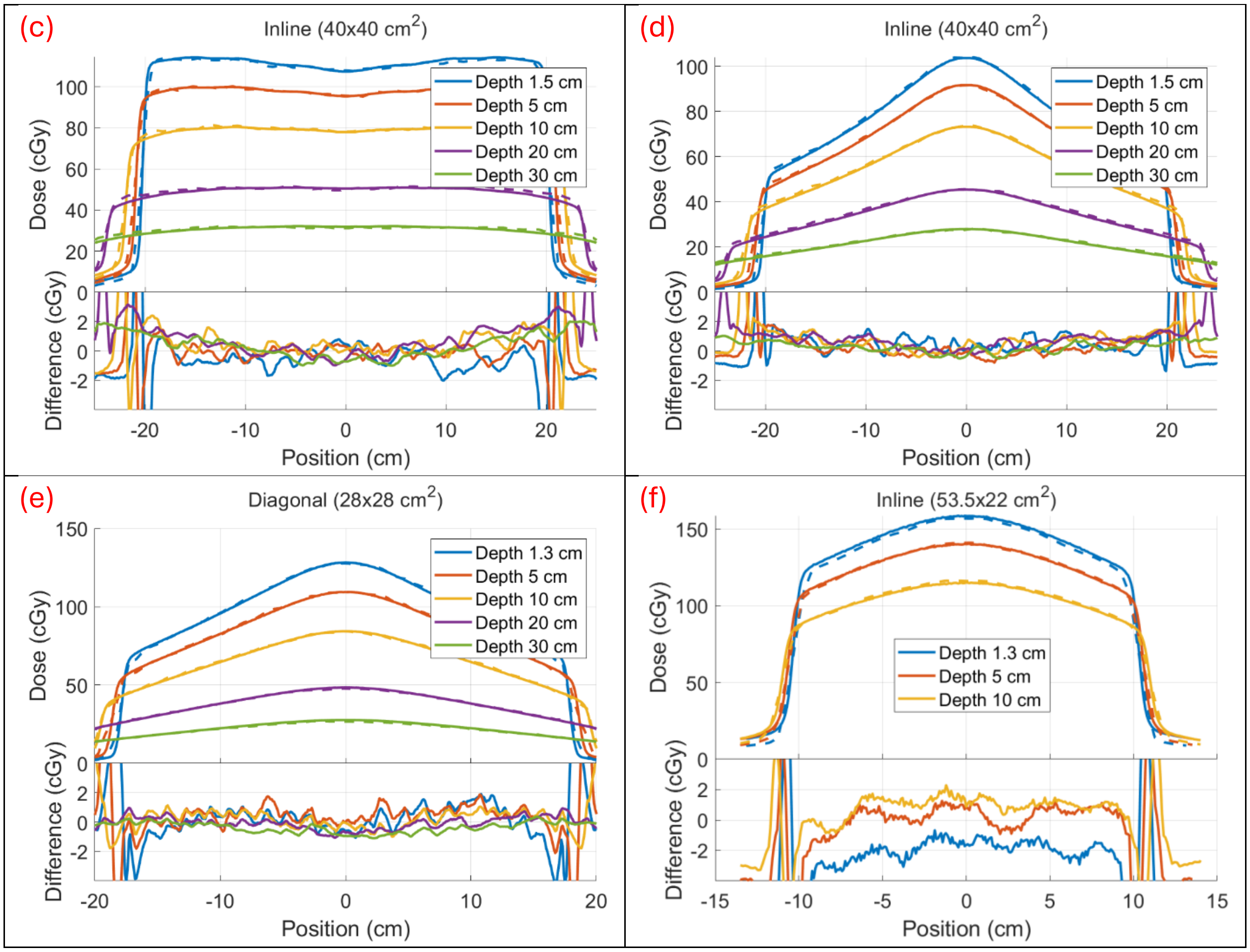

Figure S3. Comparison between calculation (dashed) and reference beams (solid) for inline and diagonal profiles of large fields. (a) TB-6X, diagonal profiles, field size 40x40 cm^2. (b) TB-6XFFF, diagonal profiles, field size 40x40 cm^2. (c) Versa-6X, inline profiles, field size 40x40 cm^2. (d) Versa-6XFFF, inline profiles, field size 40x40 cm^2. (e) Ethos, diagonal profiles, field size 28x28 cm^2. (f) Unity, inline profiles, field size 53.5x22 cm^2.

Statistical summary

Below is a statistical summary of the differences between the calculated and reference beam data. Output factors (OF) were measured/calculated at the 5 cm depth, except for Ethos and Unity, which were measured/calculated at the 10 cm depth. All values were normalized to the 10×10 cm^2 field. The OF differences for all data points were summarized using the mean, standard deviation, and maximum values for individual machines and overall (Table S1). For profile comparisons, percentage differences were calculated by normalizing to the central dose of the 10×10 cm^2 field at a depth of 1.5 cm, except for Ethos and Unity, which were normalized at 5 cm and 10 cm depths, respectively. For percent depth dose (PDD) curves, differences beyond the depth of maximum dose (1.5 cm) were summarized using the mean, standard deviation, and maximum values for individual machines and overall (Table S2). For crossline, inline, and diagonal profiles, we report the

maximum differences in the high-dose region (maxH) and low-dose region (maxL), along with the maximum distance to agreement (maxD) in the 80–20% penumbra region, for individual machines and overall (Tables S3–S5).

Output factors (OF)

Table S1. OF Difference (%) normalized to the 10×10 cm^2 field.

Machine	Mean	Std	Max
TB-06X	0.1	0.5	-1.1
TB-6XFFF	0.1	0.5	1.4
Versa-06X	-0.3	0.6	-1.7
Versa-6FFF	-0.7	0.5	-1.5
Ethos	-0.1	0.5	0.8
Unity	0.0	0.3	-0.7
Overall	-0.1	0.6	-1.7

Percent depth dose (PDD)

Table S2. PDD difference (%) normalized to the 10×10 cm^2 field at 1.5 cm.

Machine	Mean	Std	Max
TB-06X	-0.1	0.4	-1.4
TB-6XFFF	-0.4	0.4	-1.8
Versa-06X	-0.1	0.3	-1.3
Versa-6FFF	-0.1	0.3	1.2
Ethos	-0.4	0.3	-1.4
Unity	0.2	0.5	1.8
Overall	-0.1	0.4	-1.8

Cross profiles of the 10x10 cm^2 field

Table S3. Difference (%) of cross profiles for the 10x10 cm^2 field normalized to the depth at 1.5 cm. “maxH”: maximum difference in the high dose region. “maxL”: maximum difference in the low dose region. “maxD”: maximum distance to agreement in the 80%-20% penumbra region.

Machine	maxH (%)	maxL (%)	maxD (cm)
TB-06X	0.9	0.4	0.0
TB-6XFFF	-1.7	0.4	0.0
Versa-06X	-1.0	1.7	0.1
Versa-6FFF	1.5	0.9	0.1
Ethos	1.2	0.4	0.0
Unity	1.7	3.1	0.2
Overall	-1.7	3.1	0.2

Cross profiles of large fields

Table S4. Difference (%) of cross profiles for large fields normalized to the 10x10 cm^2 field at the depth of 1.5 cm. “maxH”: maximum difference in the high dose region. “maxL”: maximum difference in the low dose region. “maxD”: maximum distance to agreement in the 80%-20% penumbra region.

Machine	Field (cm^2)	maxH (%)	maxL (%)	maxD (cm)
TB-06X	40x40	2.7	2.2	0.2
TB-6XFFF	40x40	1.8	2.2	0.3
Versa-06X	40x40	2.5	2.8	0.2
Versa-6FFF	40x40	1.7	1.4	0.2
Ethos	28x28	1.4	0.9	0.3
Unity	53.5x22	-3.4	7.5	0.3
Overall	—	-3.4	7.5	0.3

Diagonal or inline profiles of large fields

Table S5. Difference (%) of diagonal or inline profiles for large fields normalized to the 10×10 cm^2 field at the depth of 1.5 cm. “maxH”: maximum difference in the high dose region. “maxL”: maximum difference in the low dose region. “maxD”: maximum distance to agreement in the 80%-20% penumbra region.

Machine	Profile (field cm^2)	maxH (%)	maxL (%)	maxD (cm)
TB-06X	Diagonal (40x40)	3.2	4.8	0.5
TB-6XFFF	Diagonal (40x40)	-2.1	2.5	0.5
Versa-06X	Inline (40x40)	3.2	1.9	0.3
Versa-6FFF	Inline (40x40)	1.6	1.1	0.2
Ethos	Diagonal (28x28)	-2.8	0.7	0.4
Unity	Inline (53.5x22)	-3.4	5.5	0.3
Overall	—	-3.4	5.5	0.5
