# Supplementary figures and images for: Automatic, machine‐agnostic, convolution‐based beam, and fluence modeling for Monte Carlo independent dose calculation
Source: Med Phys. 2025 Apr 14;52(7):e17822. doi: 10.1002/mp.17822 (PMC12257457; doi:10.1002/mp.17822)

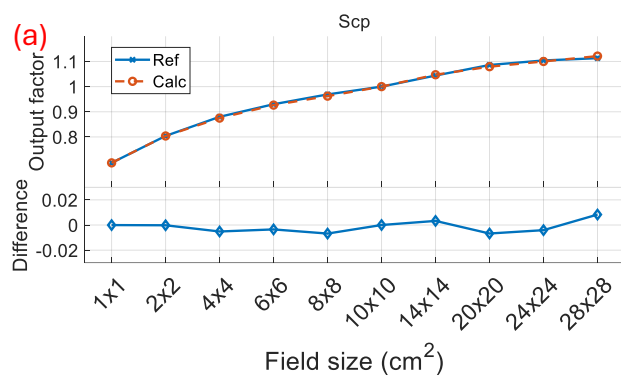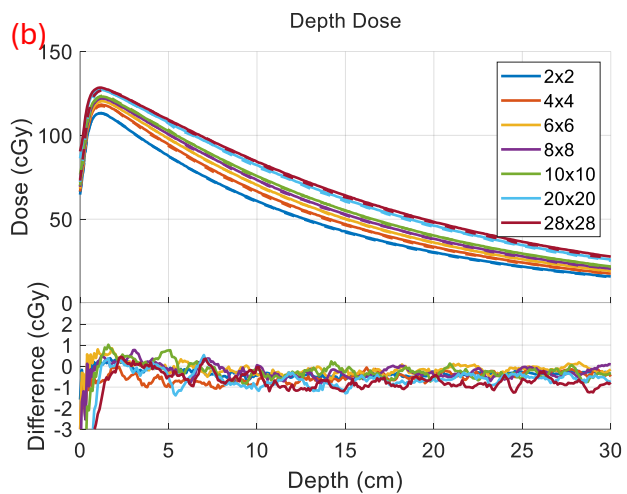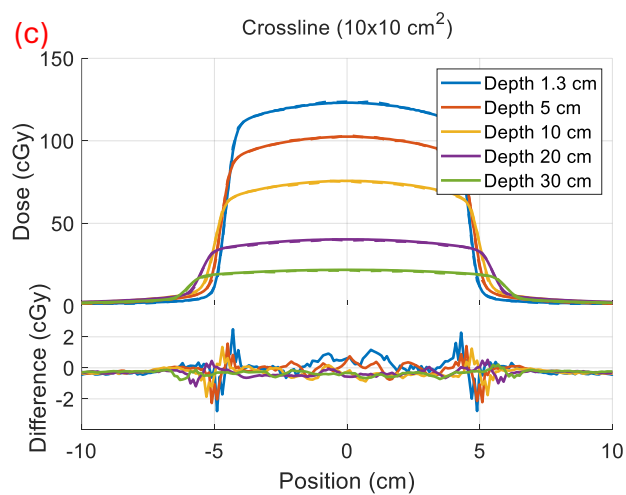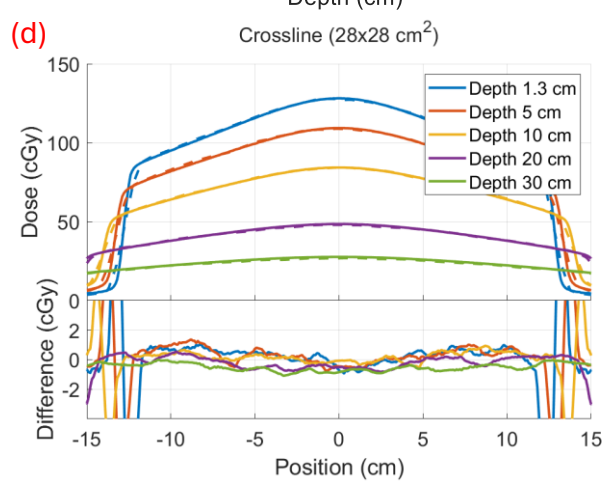

Supplement: Supplementary file 2 — Supporting information [file MP-52-0-s003.pdf]

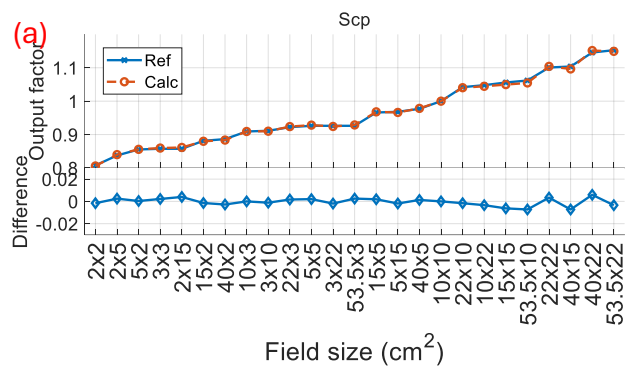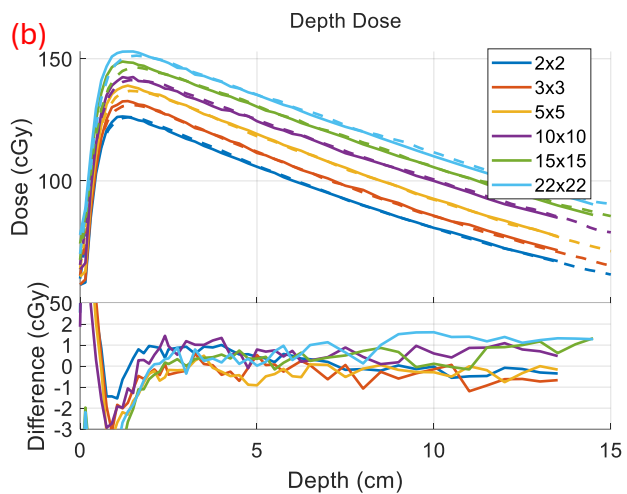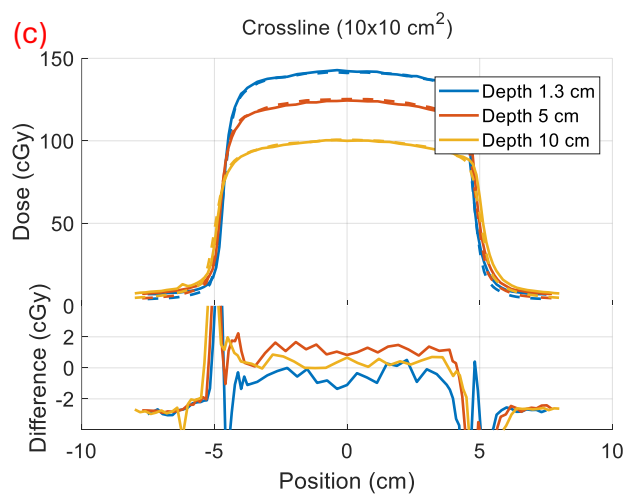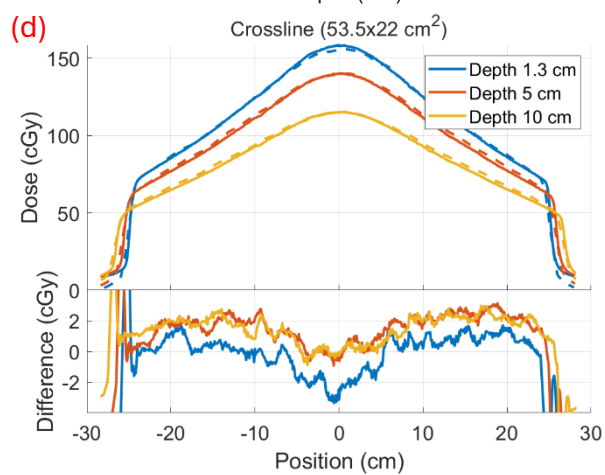

Supplement: Supplementary file 3 — Supporting information [file MP-52-0-s001.pdf]

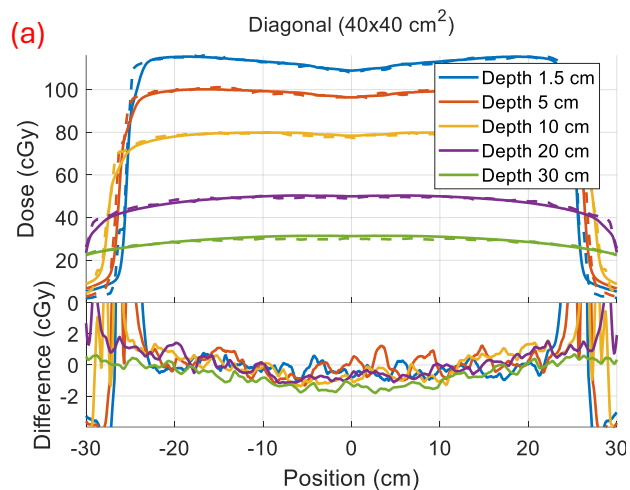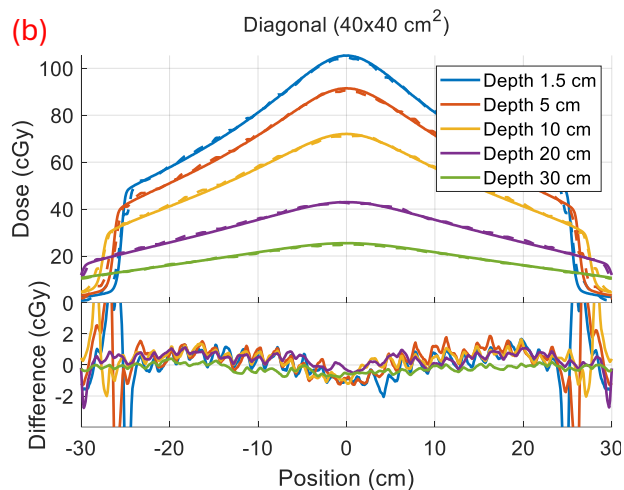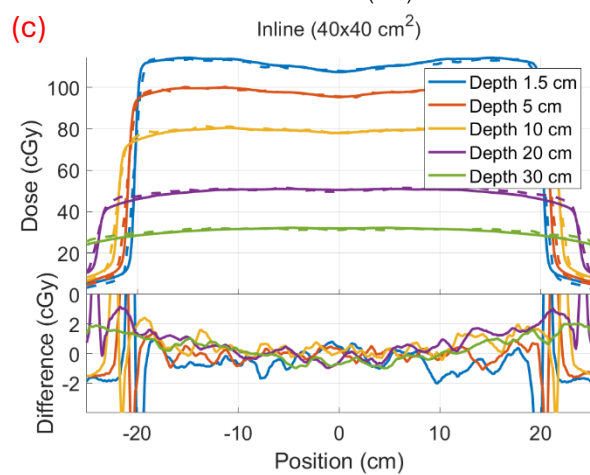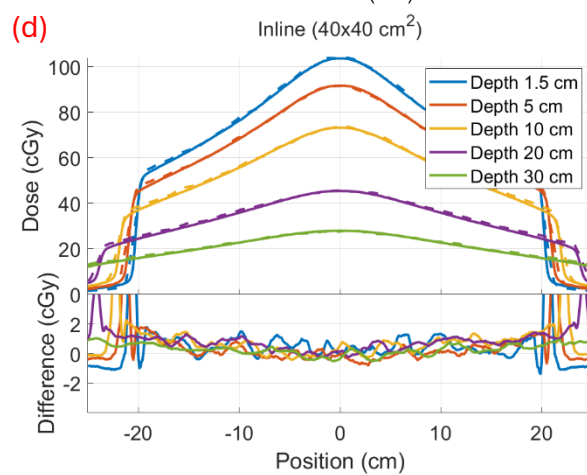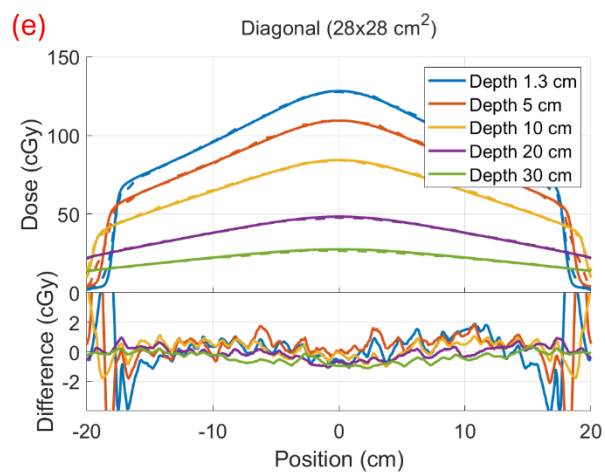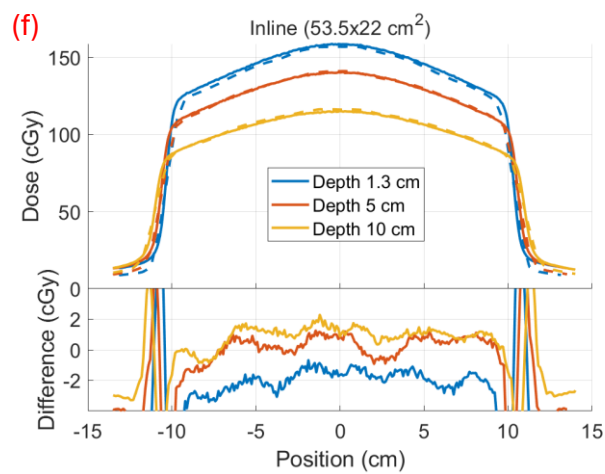

Supplement: Supplementary file 4 — Supporting information [file MP-52-0-s002.pdf]
